# The role of activation of PERK in activating glycogen synthase kinase 3(GSK-3) by oleic acid(OA) in type 2 diabetes

**DOI:** 10.1186/1687-9856-2013-S1-O35

**Published:** 2013-10-03

**Authors:** Wei Wu, Shan Huang, Xiaoping Luo

**Affiliations:** 1 Pediatrics Department, Tongji Hospital, Huazhong University of Science and Technology, China

## 

ER-stress induced apoptosis of beta cells is an important mechanism of type 2 diabetes. PERK can be activated by the overactivation of ER-stress which will induce beta cells apoptosis. This study is to reveal the role of ER-stress, GSK-3 and the potential signal pathway during beta cells apoptosis.

The alterations of ER-stress related signal factors and kinases induced by OA are assessed by western blot, and the changes of PERK and AMPK are analyzed by ELISA meanwhile. The phosphorylated GSK-3β and total GSK-3 are detected by western blot, while PERK are inhibited by the transfection of P58IPK plasmid and AMPK are inhibited by the inhibitor Compound C. Finally, the interaction between PERK and GSK-3 are identified by co- immunoprecipitation and direct immuno-fluorescence. The study shows 1. The expression of GRP78, ATF6, XBP1, PERK and AMPK significant increased in the presence of 0.4mM OA(P<0.01) after OA treatment. 2. Activity of PERK and AMPK significantly augmented in the presence of OA (P<0.01). 3. Detections of the alterations of GSK-3 after inhibiting the activity of PERK and AMPK shows (1) GSK-3β was activated when treated with OA; (2) the phosphorylation of GSK-3β obviously increased after the inhibition of PERK by transfection of P58IPK plasmid (P<0.01); (3) the activity of GSK-3 had no change after the inhibition of AMPK by Compound C.(P>0.05). 4. Co-immunoprecipitation of PERK and GSK-3 verified the association of PERK and GSK-3. 5. Obvious co-localization was observed after merging FITC-conjugated PERK and Rhodamine-conjugated GSK-3β. These results suggest activation of GSK-3 can induce beta cells apoptosis by ER-stress which is caused after long-time exposure to free fatty acids. PERK will activate GSK-3 directly. Collectively, PERK-GSK-3 signal pathway will play an important role during beta cell apoptosis in type 2 DM.

